# Sex Differences in COVID-19 Hospitalization and Hospital Mortality among Patients with COPD in Spain: A Retrospective Cohort Study

**DOI:** 10.3390/v14061238

**Published:** 2022-06-07

**Authors:** Javier de Miguel-Diez, Ana Lopez-de-Andres, Rodrigo Jimenez-Garcia, Jose M. de Miguel-Yanes, Valentin Hernández-Barrera, David Carabantes-Alarcon, Jose J. Zamorano-Leon, Marta Lopez-Herranz, Ricardo Omaña-Palanco

**Affiliations:** 1 Respiratory Department, Hospital General Universitario Gregorio Marañón, Instituto de Investigación Sanitaria Gregorio Marañón (IiSGM), Universidad Complutense de Madrid, 28007 Madrid, Spain; javier.miguel@salud.madrid.org; 2 Department of Public Health & Maternal and Child Health, Faculty of Medicine, Universidad Complutense de Madrid, 28040 Madrid, Spain; rodrijim@ucm.es (R.J.-G.); dcaraban@ucm.es (D.C.-A.); josejzam@ucm.es (J.J.Z.-L.); romana@ucm.es (R.O.-P.); 3 Internal Medicine Department, Hospital General Universitario Gregorio Marañón, Instituto de Investigación Sanitaria Gregorio Marañón (IiSGM), Universidad Complutense de Madrid, 28007 Madrid, Spain; josemaria.demiguel@salud.madrid.org; 4 Preventive Medicine and Public Health Teaching and Research Unit, Health Sciences Faculty, Universidad Rey Juan Carlos, Alcorcón, 28922 Madrid, Spain; valentin.hernandez@urjc.es; 5 Nursing Department, Faculty of Nursing, Physiotherapy and Podology, Universidad Complutense de Madrid, 28040 Madrid, Spain; martal11@ucm.es

**Keywords:** COVID-19, COPD, sex, incidence, in-hospital mortality, Spain

## Abstract

(1) Background: We aimed to assess the effect of COPD in the incidence of hospital admissions for COVID-19 and on the in-hospital mortality (IHM) according to sex. (2) Methods: We used national hospital discharge data to select persons aged ≥40 years admitted to a hospital with a diagnosis of COVID-19 in 2020 in Spain. (3) Results: The study population included 218,301 patients. Age-adjusted incidence rates of COVID-19 hospitalizations for men with and without COPD were 10.66 and 9.27 per 1000 persons, respectively (IRR 1.14; 95% CI 1.08–1.20; *p* < 0.001). The IHM was higher in men than in women regardless of the history of COPD. The COPD was associated with higher IHM among women (OR 1.09; 95% CI 1.01–1.22) but not among men. The COPD men had a 25% higher risk of dying in the hospital with COVID-19 than women with COPD (OR 1.25, 95% CI 1.1–1.42). (4) Conclusions: Sex differences seem to exist in the effect of COPD among patients suffering COVID-19. The history of COPD increased the risk of hospitalization among men but not among women, and COPD was only identified as a risk factor for IHM among women. In any case, we observed that COPD men had a higher mortality than COPD women. Understanding the mechanisms underlying these sex differences could help predict the patient outcomes and inform clinical decision making to facilitate early treatment and disposition decisions.

## 1. Introduction

The coronavirus disease 2019 (COVID-19) pandemic has resulted in a dramatic increase in pneumonia hospitalizations [1]. It has been described that 60–90% of hospitalized patients with COVID-19 have comorbidities including hypertension, diabetes, cardiovascular disease, and chronic obstructive pulmonary disease (COPD) [2].

Despite the fact that COPD patients are prone to viral exacerbations [3,4,5], and also considering the mode of transmission via droplets and the virulence of SARS-CoV-2, it was anticipated that COPD patients would be at increased risk of suffering COVID-19 [6]. However, several studies, particularly those early in the pandemic, found a lower prevalence of COPD among hospitalized patients with COVID-19 than would be expected from population prevalence [7,8]. More recent studies have shown that having COPD is an independent risk factor for hospital admission with COVID-19, although the risk is modest [9].

COPD has also been reported to independently increase the risk of severe disease or death in some series [8,10,11,12]. A recent systematic review and meta-analysis found that death is more likely to occur in hospitalized patients with COPD than in those without the disease [13]. However, other studies have shown no association of COPD with worse outcomes [14,15,16]. These findings suggest that the risks could be less than expected.

Identifying sex-associated biological determinants may be useful to optimize COVID-19 prevention and management in women and men [17]. Even if the number of COVID-19 cases appears to be equally distributed between men and women [18], it is by now well established that men have more severe manifestations of COVID-19 that women including hospitalization, ICU admission, and short-term mortality [19]. However, questions remain about the association of sex with outcomes in COPD patients hospitalized for COVID-19.

Using records of the hospital discharge database for Spain for the year 2020, in this study, we aimed to (1) assess the effect of COPD in the incidence of hospital admissions for COVID-19 according to sex; (2) analyze the effect of COPD on the in-hospital mortality (IHM) among patients hospitalized with COVID-19 according to sex; and (3) identify which sociodemographic and clinical conditions were associated with IHM among the COPD men and women hospitalized with COVID-19.

## 2. Materials and Methods

### 2.1. Study Design

We conducted a retrospective cohort study. Data were obtained from the Spanish National Hospital Discharge Database (SNHDD). The SNHDD is an administrative database managed by the Spanish Ministry of Health (SMH) that collects information from all hospitals, public and private, in Spain. According to Spanish legislation, all Spanish hospitals must fulfill and send data to the SMH with annual periodicity [20,21].

The information collected by the SNHDD is age, sex, place of residence, dates of admission and discharge, discharge destination (home, other social or medical institution, voluntary discharge, decease), primary diagnosis, secondary diagnosis (up to 19), and therapeutic and diagnostic procedures conducted during hospitalization (up to 20). Coding in the SNHDD is executed with the International Classification of Disease—10th version (ICD-10). Details on the SNHDD can be found elsewhere [20,21].

As the first cases of confirmed COVID-19 were identified in Spain in March 2020, the study period ran from 1 March 2020 to 31 December 2020.

### 2.2. Study Population and Participants

Our study population included persons aged 40 years or over admitted to a hospital with a diagnosis of COVID-19 in the last ten months of 2020. The definition of COVID-19 used is that recommended by the SMH to codify this disease using ICD10 (see Table S1) [22,23,24] during the year 2020.

We defined as the exposed cohort the patients who suffered COPD when admitted to the hospital. A participant was classified as a COPD patient if a code for COPD (ICD10 J44.0, J44.1 and J44.9) was recorded in any diagnosis position (1–20), either as a primary or secondary diagnosis, along with a “present on admission” (POA) indicator of “yes”. The POA allowed us to determine which conditions existed before the patient came to the hospital (Table S1).

The unexposed cohort included all admissions without a code for COPD.

Exclusion criteria for both cohorts were (a) missing data for age, sex, place of residence, dates of admission or discharge and discharge destination; and (b) if the same individual was admitted more than once during the study period with COVID-19, only the first episode was analyzed.

The study population was stratified by sex for description and analysis.

### 2.3. Matching

For each person with COPD (exposed cohort), we selected a non-COPD subject (un-exposed cohort) with identical sex, age, province of residence, and month of admission. If more than one unexposed person was available for an exposed case, the person with the closest date of admission was included.

### 2.4. Variables

The main outcome variables of this investigation were the incidence of hospital admission and the IHM according to the presence of COPD and sex.

To estimate the incidence of hospitalizations in the study cohorts, we used as a denominator the weighted number of persons with and without COPD in Spain, by age groups and sex, according to the self-reported prevalence of physician-diagnosed COPD among participants in the European Health Interview Survey for Spain (EHISS2020) [25].

Secondary outcome variables were admission (yes/no) to an intensive care unit (ICU), duration of stay in the ICU, and total length of hospital stay (LOHS).

Study covariates used for matching were province of residence (Spain is divided into 50 provinces) and the month of admission.

To assess the global comorbidity, the mean number of conditions included in the Charlson Comorbidity Index (CCI) was calculated using the algorithms previously proposed by Sundararajan et al. and Quan et al. [26,27].

Specific conditions analyzed were pneumonia, acute bronchitis, lower respiratory infection, acute respiratory distress syndrome (ARDS), sepsis, obesity, myocardial infarction, congestive heart failure, peripheral vascular disease, cerebrovascular disease, dementia, rheumatoid disease, mild/moderate/severe liver disease, chronic renal disease, cancer or metastatic cancer, diabetes, bronchiectasis, asthma, and pulmonary embolism. Procedures included the use of non-invasive and invasive mechanical ventilation.

To assess the severity of COPD, we analyzed the position of the COPD code in the discharge report with two categories, “First three positions” and “Last three positions” as well as the use of oxygen prior to hospital admission and the “long-term (current) use of steroids”.

The clinical conditions and therapeutic procedures analyzed, and the ICD-10 codes used to identify them are described in Table S1.

### 2.5. Statistical Methods

The incidence rates of COVID-19 admissions were calculated for both cohorts according to sex and age groups. The direct method was used to obtain age-adjusted incidence rates using the total Spanish population as a standard. Incidence rate ratios (IRR) with 95% confidence intervals (95% CI) were estimated.

The descriptive analysis was performed with the calculation of means with standard deviation (SD) or medians with interquartile range (IQR) for the quantitative variables and with absolute and relative frequencies, expressed as percentages, for the qualitative variables.

The statistical methods applied for the comparison of means, medians, and proportions between the study subpopulations were the Student’s *t* test, the Wilcoxon–Mann–Whitney test, and the chi-square test, respectively.

Multivariable logistic regression models were constructed to identify which of the study variables were independently associated with IHM in each of the study subpopulations. This statistical method was applied following the recommendations proposed by Hosmer et al. [28]. Two-way interactions were examined.

### 2.6. Sensitivity Analysis

Even if we matched for the relevant variables that are associated with IHM, the effects of other confounding variables could not be controlled. Therefore, to assess the effect of COPD on the IHM among women, men, and persons of both sexes hospitalized with COVID-19, we constructed three multivariable logistic regression models using the matched sub-populations.

Stata 14 was the software used for matching and data analysis. A *p* < 0.05 (two-tailed) was considered statistically significant.

### 2.7. Ethical Aspects

The SNHDD database can be requested from the SMH at the link in [29]. The authorities of the ministry carry out an evaluation of the proposal, and if they consider it adequate from scientific and ethical points of view, they provide the totally anonymized records. Therefore, the study protocol was not evaluated by an ethics committee and, as this is an administrative database, informed consent was not required from the participants.

## 3. Results

In Spain, according to the SNHDD, in the last ten months of the year 2020, the total number of hospital admissions with COVID-19 was 218,736. After inclusion and exclusion criteria were applied, the study population included 218,301 patients; of them, 122,269 (56.0%) were men (Table 1). The total prevalence of COPD was 5.8% (*n* = 12,712), with a significantly higher proportion among men than women (8.62% vs. 2.25%; *p* < 0.001).

**Figure 1:**
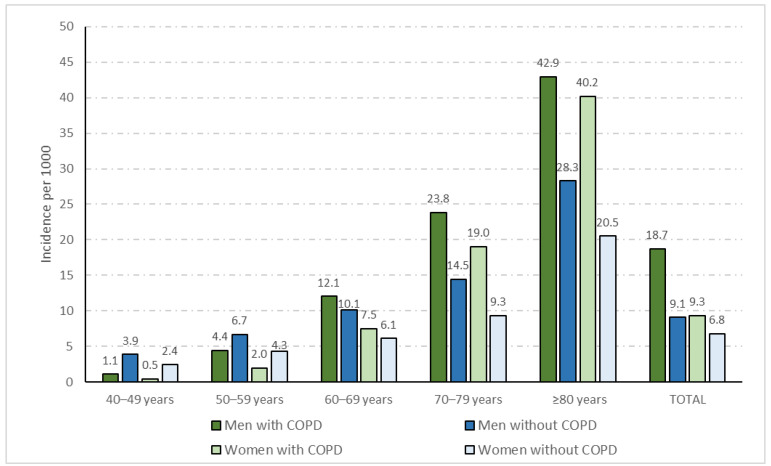
The incidence rates of hospital admission with COVID-19 per 1000 inhabitants with and without COPD according to sex and age groups.

Figure 1 shows the incidence rates for hospital admission with COVID-19 among men and women with and without COPD according to sex and age groups. The total crude incidence rates were 18.7 per 1000 men with COPD and 9.3 per 1000 women with COPD (*p* < 0.001). Incidence rates were higher among men and women with COPD than among non-COPD men and women for groups of age > 60 years. Regarding the presence of COPD, men had higher incidence rates than women in all age groups. Using the direct method, we obtained age-adjusted incidence rates for men with and without COPD of 10.66 and 9.27, respectively (IRR 1.14; 95% CI 1.08–1.20; *p* < 0.001). Among women, the age-adjusted rates were significantly lower than for men (7.27 per 1000 women with COPD and 6.78 per 1000 women without COPD; *p* < 0.001), and unlike men, the IRR for women was not significant 1.07 (95% CI 0.99–1.16; *p* = 0.098).

The distribution by sociodemographic characteristics, clinical variables, and in-hospital outcomes of patients hospitalized with COVID-19 in Spain in 2020 according to COPD status can be seen in Table 1. The proportion of men was significantly higher among those with COPD (82.99% vs. 54.34%; *p* < 0.001). COPD patients were significantly older than those without COPD (76.99 years vs. 68.81 years; *p* < 0.001).

Most COPD patients had the code for this disease within the first three positions (53.97%) and under 15% of them in the last three positions.

COPD patients had a higher mean CCI than those without COPD, with figures of 1.17 and 0.72, respectively (*p* < 0.001), and a higher prevalence of acute bronchitis, lower respiratory infection, sepsis, obesity, myocardial infarction, congestive heart failure, peripheral vascular disease, cerebrovascular disease, rheumatoid disease, mild/moderate/severe disease, chronic renal disease, cancer or metastatic cancer, and diabetes. COPD patients were found to have a higher percentage of oxygen use prior to admission (8.87% vs. 1.15%; *p* < 0.001). The long-term (current) use of steroids was recorded in 504 COPD patients (3.96%).

The use of non-invasive mechanical ventilation was codified more among COPD patients (6.82% vs. 4.48%; *p* < 0.001). However, patients with COPD less often underwent invasive mechanical ventilation than the non-COPD patients (5.24% vs. 6.66%; *p* < 0.001). The proportion of hospitalized COVID-19 patients admitted to the ICU was 7.86% for those with COPD and 9.47% for those without COPD (*p* < 0.001), and the median stay at the ICU was longer for the non-COPD patients (10 days vs. 8 days; *p* = 0.001). On the other hand, the total LOHS was higher for those with COPD (9 days vs. 8 days; *p* < 0.001). The crude IHM among the COPD population was 27.47%, decreasing significantly to 16.8% for those without this condition (*p* < 0.001).

Table 2 contains the distribution of study covariates and hospital outcomes, before and after matching, for women hospitalized for COVID-19 in Spain in 2020 according to the COPD status. Before matching, COPD women were older (76.28 years vs. 71.18 years; *p* < 0.001) and had a higher mean CCI and prevalence of all the clinical conditions described except for pneumonia (higher in non-COPD women), ARDS, sepsis, dementia, rheumatoid disease, cancer or metastatic cancer, bronchiectasis, and pulmonary embolism. The use of oxygen prior to admission, non-invasive mechanical ventilation, LOHS, and IHM (22.34% vs. 16.13%; *p* < 0.001) showed higher values among COPD women when compared to those without COPD. The distribution according to the diagnosis position of COPD was 54% in the first three positions and 16% in the last three, and 5.04% of women had long-term (current) use of steroids.

After matching the differences in the mean CCI, most chronic conditions (except in pneumonia and dementia), use of oxygen prior to admission, and non-invasive mechanical ventilation were significantly higher among COPD women. The LOHS (9 days vs. 8 days; *p* < 0.001) and the IHM (22.23% vs. 19.47%; *p* = 0.030) remained higher for women with COPD.

Table 3 shows the distribution of the study variables before and after matching for men with and without COPD. As found among women, before matching, COPD men had a higher mean age and CCI than non-COPD men (77.13 years vs. 66.82 years and 1.21 vs. 0.73; *p* < 0.001 for both). The distribution by diagnosis positions showed that around 54% and 14% of men had COPD codified in the first three and last three positions, respectively. The prevalence of every chronic condition was significantly lower among men without COPD, except for pneumonia, ARDS, and pulmonary embolism. Fewer non-COPD men received oxygen prior to admission (0.98% vs. 8.32%; *p* < 0.001) and non-invasive mechanical ventilation (5.35% vs. 7.12%; *p* < 0.001), but more non-COPD men received invasive mechanical ventilation (8.6% vs. 5.45%; *p* < 0.001). The long-term (current) use of steroids was found in 3.74%. The proportion of admission to ICU and the median days of ICU stay were significantly lower in COPD men. However, the crude IHM (28.52% vs. 17.37%; *p* < 0.001) was higher among men with COPD.

After matching, the differences between men with and without COPD narrowed and became not significant for myocardial infarction, cerebrovascular disease, rheumatoid disease, and IHM (28.41% for COPD men and 27.51% for non-COPD men). However, the lower proportion of ICU admissions remained significant among COPD men (8.17% vs. 10.08%; *p* < 0.001).

The description of IHM according to the study variables among men and women with and without COPD is shown in Table 4.

Men died in the hospital in a higher proportion than women regardless of whether they suffered COPD or not. IHM rose with age in all study subpopulations.

For both sexes, the IHM was higher when COPD was codified in the first three positions (21.01% for women and 26.61% for men) when compared to the last three positions (16.56% for women and 23.76% for men).

In all patients, regardless of sex and COPD, the use of oxygen prior to admission (IHM > 26%), the use of any mechanical ventilation (IHM > 42%), and being admitted to the ICU (IHM > 36%) were associated with higher IHM. Among women with COPD, the conditions associated with the highest mortality were sepsis (67.57%), ARDS (54.29%), and cancer or metastatic cancer (39%). These are the same most frequent chronic diseases among women without COPD. Men with sepsis and COPD had an IHM of 74.88%, and figures for ARDS, dementia, and congestive heart failure were 58.89%, 42.98%, and 40.06%, respectively. As observed among women, the mortality rates among non-COPD men according to comorbid conditions were like those found among men with COPD.

The multivariable analysis to identify the variables associated with IHM in patients hospitalized with COVID-19 in Spain, 2020, according to sex and COPD status can be seen in Table 5.

For all of the study subgroups, the risk of dying in the hospital increased as age rose and was significantly higher among those who had a code recorded for pneumonia, ARDS, sepsis, congestive heart failure, chronic renal disease, cancer or metastatic cancer, non-invasive mechanical ventilation, invasive mechanical ventilation, or admission to ICU. For men with COPD, suffering concomitant myocardial infarction, dementia, or having underwent oxygen prior to admission were risk factors for IHM. However, the diagnosis position of COPD or the long-term (current) use of steroids were not significantly associated with IHM between women and men after multivariable adjustment.

When the databases with men and women with COPD were joined, we observed that men had a 25% higher mortality risk than women (OR 1.25, 95% CI 1.1–1.42).

Finally, Table 6 shows the results of the sensitivity analysis. The multivariable logistic regression confirmed that after controlling for covariates, the presence of COPD prior to hospital admission increased the risk of dying during hospitalization among women (1.09; 95% CI 1.01–1.22), and when both sexes were analyzed together (1.07 95% CI; 1.01–1.14), but not among men.

## 4. Discussion

In this nationwide study, we found that the incidence of hospital admissions for COVID-19 was higher in COPD patients than in those without COPD, but the differences were only significant for men, and not for women. These findings are interesting because previous epidemiologic studies did not analyze the differences in the incidence rates of hospitalizations in patients with COPD and COVID-19 according to sex and, moreover, most of them failed to clearly demonstrate COPD as a risk factor for acquiring COVID-19 requiring hospitalization. In this way, it was previously suggested that COPD may be an independent risk factor for contracting COVID-19 that was not explained by other factors such as cigarette smoke or the use of inhaled corticosteroids [30].

The use of NIV was significantly higher in the COPD group than in the non-COPD group in our study. This therapeutic modality was indicated in COPD patients with COVID-19 who had acute respiratory failure if oxygen therapy failed [31]. As patients with COPD have a higher risk of developing severe COVID-19, the need for mechanical ventilator support also becomes higher in this group [32]. However, we observed that COPD patients were less often admitted to the ICU and underwent invasive mechanical ventilation than those without this condition, indicating a limited access of these patients to more advanced treatments. In this way, Gómez-Antúnez et al. [11] found that neither non-invasive ventilation, high-flow nasal cannula, or invasive mechanical ventilation were associated with better survival among COPD patients infected with COVID-19.

Our results demonstrate that the history of COPD is associated with worse prognosis, as determined by higher rates of mortality in the first group. Moreover, the IHM was higher in COPD women than in those without COPD, but we did not find differences among men with and without COPD. There are several mechanisms by which COPD may cause an increased risk of poor COVID-19 outcomes. Poor lung function reserves may lead to respiratory failure in COPD patients with superimposed COVID-19 pneumonia. Another factor that can influence is the upregulation of the SARS-CoV-2 receptor, angiotensin converting enzyme-2, in the airways and lungs of COPD patients. Over-expression of the virus receptor could allow for faster spread of the virus into the distal airways and alveoli, favoring progression of the disease. Additionally, COPD is associated with impaired innate immune responses to viruses [13]. In the same way, a Korean report using the National Health Insurance scheme also found that COPD was an independent risk factor for all-cause mortality [33]. Furthermore, in a recent meta-analysis of 39 worldwide studies, diagnosis of COPD was associated with an increase in poor clinical outcomes in patients infected with COVID-19, with increased odds of hospitalization and mortality [12]. COPD patients are frequently older and have chronic diseases, which are factors known to be associated with unfavorable outcomes of COVID-19 [34]. In fact, significant predictors of mortality in patients with COVID-19 in our study were age, diagnosis of pneumonia, ARDS, sepsis, congestive heart failure, chronic renal disease, cancer or metastatic cancer, use of mechanical ventilation (invasive and non-invasive), and admission to ICU. Furthermore, as previously pointed out by other authors, some chronic conditions were independent predictors of IHM only in one sex [35]. Thus, for men with COPD, history of myocardial infarction, dementia, or treatment with oxygen prior to admission were risk factors for IHM in our study.

We found relevant sex differences in the incidence and outcomes of COVID-19 among people with COPD. In accordance with other authors [11,32], we reported a low prevalence of COPD in patients with COVID-19. Moreover, we demonstrated that incidence rates were higher among men than women with COPD. Previous studies conducted in Spain concur that male gender was a risk factor for hospital admission due to COVID-19 among COPD patients [11,36].

When comparing mortality by sex in COPD patients, we observed that COPD men had a higher mortality risk than COPD women after adjusting for possible confounding variables. This association has been reported in Spain and other countries and for populations with COPD and other chronical conditions [11,36,37,38,39].

Sex-based differences in COVID-19 outcomes are mediated by immune response. Females have been found to show some stronger immune responses to a variety of infections, likely due to genetic and hormonal differences in the immune system [38,39,40,41].

Many components of the immune response to viral infection vary by sex. Higher levels of TLR7, an endosomal receptor expressed on dendritic and B cells, which recognizes viral infections and triggers a type I interferon (IFN) response, have been found among women than men, and this provides an advantage in response to COVID-19 [41,42]. Men have weaker T cell response than women and this is associated with worse outcomes in male patients [43]. Men also have increased cytokine concentrations and dysregulated inflammatory response, which would lead to higher complications [40]. The levels of protective SARS-CoV-2 immunoglobulin G (IgG) antibody are higher in female patients compared to male patients with severe infection, and this also contributes to better clinical outcomes [41].

Sex hormones play an important role in immune function and response to viral infections. Among women, higher estrogen levels inhibit the pro-inflammatory innate immune response, enhance T helper 2 and humoral immune response, and exert protective effects on endothelial cell function, reducing the effect of SARS-CoV-2 infections [41,43].

Among men, low testosterone levels correlate with COVID-19 severity. Males with testosterone insufficiency, as observed in elderly and comorbid patients, are predisposed to a higher systemic inflammatory response. Testosterone is critical to platelet and coagulative homeostasis; males with low testosterone levels may be predisposed to thromboembolic events in COVID-19. Testosterone deficiency may also increase angiotensin converting enzyme 2 (ACE2) receptor expression, thereby facilitating SARS-CoV-2 entry into host cells, increasing lung damage and respiratory failure [41,44]. The high prevalence of serum testosterone concentrations below the normal value has been reported among COPD men [45,46].

Smoking is more common in men than women and is thought to increase the risk for severe disease and death after COVID-19, potentially due to underlying lung disease and through the increased expression of ACE2 and the modulation of pro-inflammatory cytokines [47].

Understanding the mechanisms underlying these sex differences could help predict patient outcomes and inform clinical decision making to facilitate early treatment and disposition decisions. The recognition of sex-based differences can also help inform future research efforts to develop targeted, sex-specific therapies in COVID-19.

The main strength of our study is its population-based nature. It includes all persons aged 40 years or over admitted to a hospital with a diagnosis of COVID-19 in Spain during the study period. Furthermore, these data are subject to continuous evaluation, which supports the assumption of overall valid information. Our study, however, was not free of limitations. First, unfortunately, the SNHDD does not collect data on the Forced Expiratory Volume in 1 Second (FEV1) values, so it is not possible to provide severity based on this clinical measurement or to calculate the GOLD ABCD. However, a recent study demonstrated that COPD is an independent risk factor for all-cause mortality in COVID-19 patients, but the severity of COPD does not influence the clinical outcomes of COVID-19 [33]. In Spain, Gómez Antúnez et al. found no association between FEV1 and all-cause mortality among COPD patients with COVID-19 [11]. Furthermore, other studies using discharge databases have identified COPD as a risk factor for COVID-19 hospitalization, mortality, and other hospital outcomes without data on disease severity [48,49,50]. Second, we did not have data on the pharmacological treatment of COPD or COVID-19, which could have aided in the interpretation of the results. Third, we did not account for temporal changes in the risk of exposure to COVID-19, nor for improvements in the treatment of infected individuals over time. Fourth, the SNHDD is an administrative database that only collects age and sex as demographic variables. Therefore, the effect of other important socio-demographic characteristics such as race, educational level, social class or monthly income, among others, could not be assessed. Finally, only hospitalizations and not outpatient attendances were included. The sex differences reported in this study add essential information about the relationship between COPD and COVID-19 and may help identify effective interventions.

## 5. Conclusions

Sex differences seem to exist in the effect of COPD among patients suffering from COVID-19. In our study, the presence of COPD increased the risk of hospitalization among men but not among women, and COPD was only identified as a risk factor for IHM among women. In any case, we observed that COPD men had higher mortality than COPD women. The results reported in this study contribute important information regarding the relationship between COPD, COVID-19, and sex that may help to provide more effective interventions to protect men and women with COPD from SARS-CoV-2 infection. Future investigations should analyze the clinical and sociodemographic characteristics to confirm and explain these sex differences.

## Figures and Tables

**Table 1:** Sociodemographic and clinical characteristics and in-hospital outcomes of patients hospitalized with COVID-19 in Spain, 2020, according to chronic obstructive pulmonary disease (COPD) status.

		COPD	NO COPD	*p*-Value
N (%)		12,712 (5.8)	205,589 (94.2)	<0.001
Sex men, *n* (%)		10,550 (82.99)	111,719 (54.34)	<0.001
Age, mean (SD)		76.99 (10.21)	68.81 (14.8)	<0.001
COPD dx	First three positions, *n* (%)	6861 (53.97)	NA	NA
	Last three positions, *n* (%)	1849 (14.55)	NA	NA
40–49 years old, *n* (%)		82 (0.65)	24,638 (11.98)	<0.001
50–59 years old, *n* (%)		643 (5.06)	37,504 (18.24)	<0.001
60–69 years old, *n* (%)		2197 (17.28)	41,299 (20.09)	<0.001
70–79 years old, *n* (%)		4140 (32.57)	43,481 (21.15)	<0.001
≥80 years old, *n* (%)		5650 (44.45)	58,667 (28.54)	<0.001
CCI index, mean (SD)		1.17 (1.1)	0.72 (0.68)	<0.001
Pneumonia, *n* (%)		9600 (75.52)	163,730 (79.64)	<0.001
Acute bronchitis, *n* (%)		133 (1.05)	1564 (0.76)	<0.001
Lower respiratory infection, *n* (%)		1714 (13.48)	21,476 (10.45)	<0.001
ARDS, *n* (%)		553 (4.35)	10,101 (4.91)	0.004
Sepsis, *n* (%)		266 (2.09)	3731 (1.81)	0.023
Obesity, *n* (%)		1824 (14.35)	22,294 (10.84)	<0.001
Myocardial infarction, *n* (%)		830 (6.53)	7067 (3.44)	<0.001
Congestive heart failure, *n* (%)		2247 (17.68)	15,591 (7.58)	<0.001
Peripheral vascular disease, *n* (%)		1072 (8.43)	5902 (2.87)	<0.001
Cerebrovascular disease, *n* (%)		738 (5.81)	8432 (4.1)	<0.001
Dementia, *n* (%)		799 (6.29)	13,030 (6.34)	0.814
Rheumatoid disease, *n* (%)		302 (2.38)	4228 (2.06)	0.014
Mild/moderate/severe liver disease, *n* (%)		888 (6.99)	9630 (4.68)	<0.001
Chronic renal disease, *n* (%)		2634 (20.72)	22,458 (10.92)	<0.001
Cancer, or metastatic cancer, *n* (%)		1081 (8.5)	10,715 (5.21)	<0.001
Diabetes, *n* (%)		4113 (32.36)	47,359 (23.04)	<0.001
Bronchiectasis, *n* (%)		131 (1.03)	2192 (1.07)	0.704
Asthma, *n* (%)		567 (4.46)	10,762 (5.23)	<0.001
Pulmonary embolism, *n* (%)		272 (2.14)	5242 (2.55)	0.004
Oxygen prior to admission, *n* (%)		1127 (8.87)	2355 (1.15)	<0.001
Long-term (current) use of steroids, *n* (%)		504 (3.96)	NA	NA
Non-invasive mechanical ventilation, *n* (%)		867 (6.82)	9220 (4.48)	<0.001
Invasive mechanical ventilation, *n* (%)		666 (5.24)	13,683 (6.66)	<0.001
Admission to ICU, *n* (%)		999 (7.86)	19,467 (9.47)	<0.001
Days in ICU, median (IQR)		8 (15)	10 (16)	0.001
LOHS, median (IQR)		9 (10)	8 (8)	<0.001
IHM, *n* (%)		3492 (27.47)	34,544 (16.8)	<0.001

COPD dx: diagnosis position of the COPD code in the discharge report; CCI: Charlson Comorbidity Index; ARDS: acute respiratory distress syndrome; ICU: intensive care unit; LOHS: length of hospital stay; IHM: in-hospital mortality; NA: Not available

**Table 2:** The distribution of the study covariates and hospital outcomes of women with and without chronic obstructive pulmonary disease (COPD) with COVID-19 in Spain, 2020, before and after matching.

		Before Matching			After Matching *		
		COPD	No COPD	*p*-Value	COPD	No COPD	*p*-Value
N		2162	93,870	<0.001	2162	2162	NA
Age, mean (SD)		76.28 (11.8)	71.18 (15.15)	<0.001	76.12 (11.59)	76.12 (11.59)	NA
COPD dx	First three positions, *n* (%)	1172 (54.21)	NA	NA	1104 (54.55)	NA	NA
	Last three positions, *n* (%)	353 (16.33)	NA	NA	326 (16.11)	NA	NA
40–49 years old, *n* (%)		21 (0.97)	9536 (10.16)	<0.001	21 (1.04)	21 (1.04)	NA
50–59 years old, *n* (%)		183 (8.46)	14,853 (15.82)	<0.001	167 (8.25)	167 (8.25)	NA
60–69 years old, *n* (%)		435 (20.12)	16,427 (17.5)	<0.001	406 (20.06)	406 (20.06)	NA
70–79 years old, *n* (%)		569 (26.32)	19,359 (20.62)	<0.001	543 (26.83)	543 (26.83)	NA
≥80 years old, *n* (%)		954 (44.13)	33,695 (35.9)	<0.001	887 (43.82)	887 (43.82)	NA
CCI index, mean (SD)		1.01 (0.9)	0.7 (0.65)	<0.001	1.01 (0.9)	0.8 (0.74)	<0.001
Pneumonia, *n* (%)		1505 (69.61)	71,699 (76.38)	<0.001	1423 (70.31)	1532 (75.69)	<0.001
Acute bronchitis, *n* (%)		21 (0.97)	564 (0.6)	0.029	19 (0.94)	18 (0.89)	0.869
Lower respiratory infection, *n* (%)		332 (15.36)	10,904 (11.62)	<0.001	304 (15.02)	242 (11.96)	0.004
ARDS, *n* (%)		77 (3.56)	3387 (3.61)	0.908	70 (3.46)	70 (3.46)	0.999
Sepsis, *n* (%)		38 (1.76)	1233 (1.31)	0.074	37 (1.83)	24 (1.19)	0.094
Obesity, *n* (%)		423 (19.57)	11578 (12.33)	<0.001	392 (19.37)	257 (12.7)	<0.001
Myocardial infarction, *n* (%)		77 (3.56)	1789 (1.91)	<0.001	71 (3.51)	46 (2.27)	0.019
Congestive heart failure, *n* (%)		433 (20.03)	8446 (9)	<0.001	404 (19.96)	204 (10.08)	<0.001
Peripheral vascular disease, *n* (%)		92 (4.26)	1522 (1.62)	<0.001	87 (4.3)	42 (2.08)	<0.001
Cerebrovascular disease, *n* (%)		113 (5.23)	3759 (4)	0.004	105 (5.19)	101 (4.99)	0.775
Dementia, *n* (%)		181 (8.37)	8333 (8.88)	0.414	165 (8.15)	228 (11.26)	0.001
Rheumatoid disease, *n* (%)		74 (3.42)	2797 (2.98)	0.232	69 (3.41)	65 (3.21)	0.725
Mild/moderate/severe liver disease, *n* (%)		112 (5.18)	3667 (3.91)	0.003	106 (5.24)	68 (3.36)	0.003
Chronic renal disease, *n* (%)		410 (18.96)	10,319 (10.99)	<0.001	393 (19.42)	259 (12.8)	<0.001
Cancer, or metastatic cancer, *n* (%)		104 (4.81)	4117 (4.39)	0.341	100 (4.94)	102 (5.04)	0.885
Diabetes, *n* (%)		552 (25.53)	20377 (21.71)	<0.001	522 (25.79)	491 (24.26)	0.261
Bronchiectasis, *n* (%)		24 (1.11)	1018 (1.08)	0.910	22 (1.09)	24 (1.19)	0.767
Asthma, *n* (%)		239 (11.05)	6959 (7.41)	<0.001	220 (10.87)	137 (6.77)	<0.001
Pulmonary embolism, *n* (%)		55 (2.54)	2077 (2.21)	0.301	53 (2.62)	46 (2.27)	0.476
Oxygen prior to admission, *n* (%)		249 (11.52)	1255 (1.34)	<0.001	234 (11.56)	24 (1.19)	<0.001
Long-term (current) use of steroids, *n* (%)		109 (5.04)	NA	NA	103 (5.09)	NA	NA
Non-invasive mechanical ventilation, *n* (%)		116 (5.37)	3238 (3.45)	<0.001	110 (5.43)	59 (2.92)	<0.001
Invasive mechanical ventilation, *n* (%)		91 (4.21)	4078 (4.34)	0.760	87 (4.3)	78 (3.85)	0.474
Admission to ICU, *n* (%)		145 (6.71)	6014 (6.41)	0.573	135 (6.67)	109 (5.39)	0.086
Days in ICU, median (IQR)		8 (13)	9 (15)	0.187	8 (13)	11 (17)	0.187
LOHS, median (IQR)		9 (10)	8 (9)	<0.001	9 (10)	8 (9)	<0.001
IHM, *n* (%)		483 (22.34)	15,138 (16.13)	<0.001	450 (22.23)	394 (19.47)	0.030

* Matching was realized 1 by 1 by age, place of residence and month of admission; COPD dx: diagnosis position of the COPD code in the discharge report; CCI: Charlson Comorbidity Index; ARDS: acute respiratory distress syndrome; ICU: intensive care unit; LOHS: length of hospital stay; IHM: in-hospital mortality; NA: Not available.

**Table 3:** The distribution of study covariates and hospital outcomes of men with and without chronic obstructive pulmonary disease (COPD) with COVID-19 in Spain, 2020, before and after matching.

		Before Matching			After Matching *		
		COPD	No COPD	*p*-Value	COPD	No COPD	*p*-Value
N		10,550	11,1719	<0.001	10,550	10,550	NA
Age, mean (SD)		77.13 (9.85)	66.82 (14.2)	<0.001	76.93 (9.69)	76.93 (9.69)	NA
COPD dx	First three positions, *n* (%)	5689 (53.92)	NA	NA	5385 (54.22)	NA	NA
	Last three positions, *n* (%)	1496 (14.18)	NA	NA	1406 (14.16)	NA	NA
40–49 years old, *n* (%)		61 (0.58)	15,102 (13.52)	<0.001	52 (0.52)	52 (0.52)	NA
50–59 years old, *n* (%)		460 (4.36)	22,651 (20.27)	<0.001	432 (4.35)	432 (4.35)	NA
60–69 years old, *n* (%)		1762 (16.7)	24,872 (22.26)	<0.001	1697 (17.09)	1697 (17.09)	NA
70–79 years old, *n* (%)		3571 (33.85)	24,122 (21.59)	<0.001	3404 (34.27)	3404 (34.27)	NA
≥80 years old, *n* (%)		4696 (44.51)	24,972 (22.35)	<0.001	4347 (43.77)	4347 (43.77)	NA
CCI index, mean (SD)		1.21 (1.11)	0.73 (0.66)	<0.001	1.21 (1.11)	1.01 (0.96)	<0.001
Pneumonia, *n* (%)		8095 (76.73)	92,031 (82.38)	<0.001	7677 (77.3)	8070 (81.25)	<0.001
Acute bronchitis, *n* (%)		112 (1.06)	1000 (0.9)	0.085	100 (1.01)	80 (0.81)	0.134
Lower respiratory infection, *n* (%)		1382 (13.1)	10,572 (9.46)	<0.001	1282 (12.91)	998 (10.05)	<0.001
ARDS, *n* (%)		476 (4.51)	6714 (6.01)	<0.001	450 (4.53)	605 (6.09)	<0.001
Sepsis, *n* (%)		228 (2.16)	2498 (2.24)	0.619	215 (2.16)	273 (2.75)	0.008
Obesity, *n* (%)		1401 (13.28)	10,716 (9.59)	<0.001	1329 (13.38)	782 (7.87)	<0.001
Myocardial infarction, *n* (%)		753 (7.14)	5278 (4.72)	<0.001	709 (7.14)	684 (6.89)	0.487
Congestive heart failure, *n* (%)		1814 (17.19)	7145 (6.4)	<0.001	1685 (16.97)	1016 (10.23)	<0.001
Peripheral vascular disease, *n* (%)		980 (9.29)	4380 (3.92)	<0.001	936 (9.42)	633 (6.37)	<0.001
Cerebrovascular disease, *n* (%)		625 (5.92)	4673 (4.18)	<0.001	580 (5.84)	627 (6.31)	0.163
Dementia, *n* (%)		618 (5.86)	4697 (4.2)	<0.001	577 (5.81)	765 (7.7)	<0.001
Rheumatoid disease, *n* (%)		228 (2.16)	1431 (1.28)	<0.001	217 (2.18)	197 (1.98)	0.321
Mild/moderate/severe liver disease, *n* (%)		776 (7.36)	5963 (5.34)	<0.001	723 (7.28)	489 (4.92)	<0.001
Chronic renal disease, *n* (%)		2224 (21.08)	12,139 (10.87)	<0.001	2100 (21.14)	1651 (16.62)	<0.001
Cancer, or metastatic cancer, *n* (%)		977 (9.26)	6598 (5.91)	<0.001	923 (9.29)	820 (8.26)	0.010
Diabetes, *n* (%)		3561 (33.75)	26,982 (24.15)	<0.001	3357 (33.8)	3012 (30.33)	<0.001
Bronchiectasis, *n* (%)		107 (1.01)	1174 (1.05)	0.724	103 (1.04)	151 (1.52)	0.002
Asthma, *n* (%)		328 (3.11)	3803 (3.4)	0.109	309 (3.11)	272 (2.74)	0.119
Pulmonary embolism, *n* (%)		217 (2.06)	3165 (2.83)	<0.001	212 (2.13)	293 (2.95)	<0.001
Oxygen prior to admission, *n* (%)		878 (8.32)	1100 (0.98)	<0.001	809 (8.15)	128 (1.29)	<0.001
Long-term (current) use of steroids, *n* (%)		395 (3.74)	NA	NA	376 (3.79)	NA	NA
Non-invasive mechanical ventilation, *n* (%)		751 (7.12)	5982 (5.35)	<0.001	720 (7.25)	601 (6.05)	0.001
Invasive mechanical ventilation, *n* (%)		575 (5.45)	9605 (8.6)	<0.001	550 (5.54)	714 (7.19)	<0.001
Admission to ICU, *n* (%)		854 (8.09)	13,453 (12.04)	<0.001	811 (8.17)	1001 (10.08)	<0.001
Days in ICU, median (IQR)		8 (15)	11 (17)	<0.001	8 (15)	11 (19)	0.001
LOHS, median (IQR)		9 (10)	8 (9)	0.076	9 (10)	9 (10)	0.787
IHM, *n* (%)		3009 (28.52)	19,406 (17.37)	<0.001	2822 (28.41)	2732 (27.51)	0.155

* Matching was realized 1 by 1 by age, place of residence, and month of admission; COPD dx: diagnosis position of the COPD code in the discharge report; CCI: Charlson Comorbidity Index; ARDS: acute respiratory distress syndrome; ICU: intensive care unit; LOHS: length of hospital stays; IHM: in-hospital mortality; NA: Not available.

**Table 4:** The in-hospital mortality after matching for women and men with COVID-19 in Spain, 2020, according to chronic obstructive pulmonary disease (COPD) status.

		Women			Men		
		COPD	No COPD	*p*-Value	COPD	No COPD	*p*-Value
N (%)		450 (22.23)	394 (19.47)	0.030	2822 (28.41)	2732 (27.51)	0.155
Age, mean (SD)		80.68 (10.58)	83.35 (9.19)	<0.001	80.29 (8.54)	81.28 (7.84)	<0.001
COPD dx	First three positions, *n* (%)	232 (21.01)	NA	NA	1433 (26.61)	NA	NA
	Last three positions, *n* (%)	54 (16.56)	NA	NA	334 (23.76)	NA	NA
40–49 years old, *n* (%)		2 (9.52)	0 (0)	NA	2 (3.85)	1 (1.92)	0.566
50–59 years old, *n* (%)		19 (11.38)	6 (3.59)	0.010	52 (12.04)	30 (6.94)	0.012
60–69 years old, *n* (%)		53 (13.05)	31 (7.64)	0.012	273 (16.09)	205 (12.08)	0.001
70–79 years old, *n* (%)		99 (18.23)	74 (13.63)	0.039	850 (24.97)	748 (21.97)	0.004
≥80 years old, *n* (%)		277 (31.23)	283 (31.91)	0.759	1645 (37.84)	1748 (40.21)	0.024
CCI index, mean (SD)		1.42 (1.15)	1.11 (0.95)	<0.001	1.42 (1.15)	1.29 (1.12)	<0.001
Pneumonia, *n* (%)		348 (24.46)	319 (20.82)	0.018	2385 (31.07)	2307 (28.59)	0.001
Acute bronchitis, *n* (%)		6 (31.58)	1 (5.56)	0.071	21 (21)	17 (21.25)	0.967
Lower respiratory infection, *n* (%)		63 (20.72)	44 (18.18)	0.458	302 (23.56)	266 (26.65)	0.090
ARDS, *n* (%)		38 (54.29)	38 (54.29)	0.999	265 (58.89)	329 (54.38)	0.144
Sepsis, *n* (%)		25 (67.57)	16 (66.67)	0.942	161 (74.88)	201 (73.63)	0.753
Obesity, *n* (%)		87 (22.19)	46 (17.9)	0.186	374 (28.14)	169 (21.61)	0.001
Myocardial infarction, *n* (%)		20 (28.17)	12 (26.09)	0.805	248 (34.98)	251 (36.7)	0.504
Congestive heart failure, *n* (%)		147 (36.39)	61 (29.9)	0.112	675 (40.06)	434 (42.72)	0.174
Peripheral vascular disease, *n* (%)		28 (32.18)	5 (11.9)	0.018	295 (31.52)	197 (31.12)	0.868
Cerebrovascular disease, *n* (%)		27 (25.71)	22 (21.78)	0.508	192 (33.1)	231 (36.84)	0.174
Dementia, *n* (%)		44 (26.67)	82 (35.96)	0.052	248 (42.98)	353 (46.14)	0.249
Rheumatoid disease, *n* (%)		13 (18.84)	13 (20)	0.865	76 (35.02)	57 (28.93)	0.186
Mild/moderate/severe liver disease, *n* (%)		28 (26.42)	15 (22.06)	0.516	168 (23.24)	137 (28.02)	0.060
Chronic renal disease, *n* (%)		134 (34.1)	68 (26.25)	0.035	784 (37.33)	634 (38.4)	0.503
Cancer, or metastatic cancer, *n* (%)		39 (39)	41 (40.2)	0.862	331 (35.86)	297 (36.22)	0.876
Diabetes, *n* (%)		150 (28.74)	113 (23.01)	0.038	947 (28.21)	875 (29.05)	0.459
Bronchiectasis, *n* (%)		6 (27.27)	6 (25)	0.861	24 (23.3)	36 (23.84)	0.921
Asthma, *n* (%)		44 (20)	20 (14.6)	0.197	66 (21.36)	58 (21.32)	0.992
Pulmonary embolism, *n* (%)		13 (24.53)	12 (26.09)	0.859	70 (33.02)	87 (29.69)	0.426
Oxygen prior to admission, *n* (%)		62 (26.5)	12 (50)	0.019	279 (34.49)	40 (31.25)	0.473
Long-term (current) use of steroids, *n* (%)		24 (23.30)	NA	NA	86 (22.87)	NA	NA
Non-invasive mechanical ventilation, *n* (%)		50 (45.45)	31 (52.54)	0.380	341 (47.36)	289 (48.09)	0.793
Invasive mechanical ventilation, *n* (%)		42 (48.28)	33 (42.31)	0.442	332 (60.36)	380 (53.22)	0.011
Admission to ICU, *n* (%)		62 (45.93)	39 (35.78)	0.110	411 (50.68)	450 (44.96)	0.015
Days in ICU, median (IQR)		8 (12)	15 (22)	0.004	9 (14)	13 (20)	<0.001
LOHS, median (IQR)		8 (10)	7 (9)	0.040	8 (10)	7 (10)	0.892

COPD dx: diagnosis position of the COPD code in the discharge report; CCI: Charlson Comorbidity Index; ARDS: acute respiratory distress syndrome; ICU: intensive care unit; LOHS: length of hospital stay; NA: Not available.

**Table 5:** The multivariable analysis of factors associated with in-hospital mortality after matching for men and women with COVID-19 in Spain, 2020, according to chronic obstructive pulmonary disease (COPD) status.

	COPD Women	COPD Men	COPD Both Sex	No COPD Women	No COPD Men	No COPD Both Sex
	OR (95%)	OR (95%)	OR (95%)	OR (95%)	OR (95%)	OR (95%)
Age 40–49 years	1	1	1	NAC	1	1
Age 50–59 years	1.14 (0.21–6.14)	3 (0.63–14.29)	1.96 (0.64–6.02)	1	4.01 (0.5–31.98)	4.59 (0.58–36.03)
Age 60–69 years	0.96 (0.19–4.96)	5.09 (1.1–23.58)	2.76 (0.92–8.27)	2.13 (0.78–5.84)	6.15 (0.8–47.41)	7.36 (0.96–56.2)
Age 70–79 years	2 (0.39–10.21)	10.2 (2.21–47.06)	5.47 (1.84–16.3)	5.87 (2.22–15.54)	15.74 (2.05–120.79)	18.88 (2.48–143.77)
Age ≥80 years	4.79 (0.94–24.47)	22.85 (4.95–105.47)	12.39 (4.15–36.93)	24.46 (9.25–64.73)	48.79 (6.35–374.74)	59.73 (7.84–454.98)
Pneumonia	1.89 (1.37–2.61)	1.91 (1.65–2.21)	1.9 (1.67–2.17)	1.99 (1.37–2.9)	1.53 (1.31–1.78)	1.59 (1.38–1.83)
ARDS	2.34 (1.29–4.23)	2.46 (1.96–3.08)	2.4 (1.94–2.96)	4.4 (2.36–8.21)	2.33 (1.91–2.86)	2.49 (2.05–3.01)
Sepsis	5.2 (2.34–11.56)	4.67 (3.32–6.58)	4.7 (3.44–6.43)	6.06 (2.2–16.74)	4.69 (3.45–6.39)	4.78 (3.56–6.42)
MI	-	1.3 (1.09–1.54)	1.26 (1.07–1.49)	1.24 (1.06–1.46)	1.42 (1.18–1.7)	1.39 (1.17–1.66)
CHF	1.8 (1.37–2.36)	1.57 (1.4–1.78)	1.61 (1.44–1.8)	1.47 (11.8–2.05)	1.49 (1.29–1.73)	1.43 (1.25–1.64)
CVD	-	-	-	1.35 (1.17–1.72)	1.45 (1.21–1.75)	1.38 (1.16–1.64)
Dementia	-	1.9 (1.58–2.28)	1.71 (1.45–2.02)	1.9 (1.37–2.65)	2.03 (1.72–2.38)	1.98 (1.71–2.29)
MMSLD	-	-	-	-	1.27 (1.01–1.6)	1.26 (1.01–1.57)
CRD	1.52 (1.16–2.01)	1.42 (1.27–1.59)	1.43 (1.29–1.59)	1.33 (1.12–1.57)	1.44 (1.28–1.63)	1.38 (1.23–1.55)
CMC	3.62 (2.23–5.88)	1.87 (1.6–2.19)	1.98 (1.71–2.3)	5.74 (3.48–9.46)	1.76 (1.49–2.08)	1.98 (1.69–2.31)
Diabetes	-	-	-	1.11 (1.05–1.18)	1.06 (1.02–1.12)	1.07 (1.03–1.11)
OPA	-	1.48 (1.26–1.75)	1.43 (1.23–1.66)	-	-	-
NIMV	3.12 (1.97–4.97)	2.34 (1.96–2.79)	2.41 (2.04–2.84)	5.26 (2.76–10.02)	2.77 (2.28–3.37)	2.93 (2.43–3.53)
IMV	1.88 (1.05–3.05)	3.86 (2.95–5.04)	3.46 (2.7–4.44)	3.8 (1.57–9.21)	3.84 (2.92–5.05)	3.78 (2.91–4.91)
Admission ICU	2.75 (1.56–4.85)	1.6 (1.27–2)	1.72 (1.4–2.12)	1.72 (1.15–2.10)	1.39 (1.1–1.76)	1.41 (1.12–1.77)
Male sex	NA	NA	1.25 (1.1–1.42)	NA	NA	1.31 (1.15–1.5)

Only significant OR are shown in the table. ARDS: acute respiratory distress syndrome; MI: myocardial infarction; CHF: congestive heart failure; CVD: cerebrovascular disease; MMSLD: mild/moderate/severe liver disease; CRD: chronic renal disease; CMC: cancer or metastatic cancer; OPA: oxygen prior to admission; NIMV: non-invasive mechanical ventilation; IMV: invasive mechanical ventilation; ICU: intensive care unit; NS: non-significant; NA: not applicable; NAC (not available categories due to no in hospital mortality in this category).

**Table 6:** Multivariable analysis of factors associated with in-hospital mortality after matching for patients with COVID-19 in Spain, 2020, according to chronic obstructive pulmonary disease (COPD) status.

	All Women	All Men	Both Sex
	OR (95%)	OR (95%)	OR (95%)
Age 40–49 years	1	1	1
Age 50–59 years	1.18 (0.25–5.54)	3.53 (1.03–12.16)	2.54 (0.96–6.7)
Age 60–69 years	1.29 (0.29–5.82)	5.61 (1.67–18.91)	3.72 (1.44–9.61)
Age 70–79 years	2.9 (0.65–12.95)	12.45 (3.7–41.86)	8.19 (3.17–21.12)
Age ≥80 years	8.82 (1.98–39.29)	32.64 (9.7–109.78)	21.79 (8.44–56.22)
Pneumonia	1.91 (1.5–2.44)	1.71 (1.54–1.9)	1.74 (1.58–1.92)
ARDS	3.04 (2–4.62)	2.37 (2.04–2.75)	2.43 (2.11–2.8)
Sepsis	5.66 (3.04–10.54)	4.63 (3.69–5.81)	4.72 (3.82–5.85)
Myocardial infarction	1.25 (1.17–1.49)	1.35 (1.19–1.53)	1.33 (1.18–1.5)
Congestive heart failure	1.54 (1.24–1.91)	1.52 (1.4–1.69)	1.53 (1.41–1.67)
Cerebrovascular disease	1.29 (1.11–1.77)	1.32 (1.16–1.51)	1.30 (1.14–1.46)
Dementia	1.57 (1.23–2.01)	1.98 (1.75–2.23)	1.88 (1.68–2.09)
Mild/Moderate/severe liver disease	-	1.14 (1.04–1.31)	1.10 (1.01–1.20)
Chronic renal disease,	1.28 (1.03–1.58)	1.42 (1.3–1.54)	1.4 (1.29–1.51)
Cancer or metastatic cancer	4.4 (3.12–6.19)	1.83 (1.63–2.05)	1.99 (1.78–2.21)
Diabetes	1.10 (1.04–1.17)	1.07 (1.03–1.14)	1.08 (1.05–1.11)
Non-invasive mechanical ventilation	3.81 (2.63–5.52)	2.54 (2.23–2.89)	2.65 (2.35–3)
Invasive mechanical ventilation	2.31 (1.35–3.94)	3.7 (3.06–4.48)	3.49 (2.92–4.17)
Admission to ICU	2.28 (1.44–3.61)	1.49 (1.26–1.75)	1.56 (1.34–1.81)
Male sex	NA	NA	1.27 (1.16–1.4)
COPD	1.09 (1.01–1.22)	-	1.07 (1.01–1.14)

Only significant OR are shown in the table. COPD: chronic obstructive pulmonary disease; ARDS: acute respiratory distress syndrome; ICU: intensive care unit; NS: non-significant; NA: not applicable.

## Data Availability

According to the contract signed with the Spanish Ministry of Health and Social Services, which provided access to the databases from the Spanish National Hospital Database (RAE-CMBD, Registro de Actividad de Atención Especializada. Conjunto Mínimo Básico de Datos, Registry of Specialized Health Care Activities. Minimum Basic Dataset), we cannot share the databases with any other investigator, and we have to destroy the databases once the investigation has concluded. Consequently, we cannot upload the databases to any public repository. However, any investigator can apply for access to the databases by filling out the questionnaire available at http://www.msssi.gob.es/estadEstudios/estadisticas/estadisticas/estMinisterio/SolicitudCMBDdocs/Formulario_Peticion_Datos_CMBD.pdf. (accessed on 15 April 2022). All other relevant data are included in the paper.

## Supplementary Materials

The following supporting information can be downloaded at: https://www.mdpi.com/article/10.3390/v14061238/s1, Table S1: ICD-10 codes for diagnosis and therapeutic procedures used in this investigation.
