# Supplementary material for: Sex Differences in COVID-19 Hospitalization and Hospital Mortality among Patients with COPD in Spain: A Retrospective Cohort Study
Source: Viruses. 2022 Jun 7;14(6):1238. doi: 10.3390/v14061238 (PMC9229343; doi:10.3390/v14061238)
Supplement: Supplementary file 1 [file viruses-14-01238-s001.zip › viruses-1664684-supplementary.pdf]

Table S1. ICD-10 codes for diagnosis and therapeutic procedures used in this investigation.

Description	ICD-10 codes
COVID 19	B34.2
	B97.29
	U07.1
Chronic Obstructive Pulmonary Disease	J44.0, J44.1 and J44.9
Pneumonia	J12.82, J12.89, J12.9
Acute bronchitis	J20.8, J20.9
Lower respiratory infection	J22, J98.8
Acute respiratory distress syndrome (ARDS)	J80
Sepsis	R65.20, R65.21
Obesity	E66.09, E66.1, E66.2, E66.01, E66.3, E66.8 E66.9
Bronchiectasis	J47
Asthma	J45
Pulmonary embolism	I26.xx
Oxygen prior to admission	Z99.81
Long term (current) use of steroids.	Z79.5
Non-invasive mechanical ventilation	5A09357, 5A09457, 5A09557
Invasive mechanical ventilation	5A1945Z, 5A1955Z, 5A1935Z

*Each discharge diagnosis has a "Present on Admission (POA)" indicator assigned according to the ICD-10-CM Official Guidelines for Coding and Reporting (https://icdlist.com/icd-10/guidelines/). The reporting options and definitions for POA are "Y" (present at admission); "N" (not present at admission); "U" (lack documentation to determine presence at admission); "W" (provider is unable to clinically determine if the condition was present); and unreported/not used. Only COPD patients with "Y" were included in the study population.

The ICD 10 codes for conditions included in the Charlson Comorbidity Index can be found in references:

Sundararajan V, Henderson T, Perry C, Muggivan A, Quan H, Ghali WA. New ICD-10 version of the Charlson comorbidity index predicted in-hospital mortality. J. Clin. Epidemiol 2004;57:1288–94. doi: 10.1016/j.jclinepi.2004.03.012.

Quan H, Sundararajan V, Halfon P, Fong A, Burnand B, Luthi JC, et al. Coding algorithms for defining comorbidities in ICD-9-CM and ICD-10 administrative data. Med Care. 2005;43:1130-9. doi: 10.1097/01.mlr.0000182534.19832.83.
